# Pediatric Bed Capacity, Bed Strain, and Load Imbalance During the 2022 Respiratory Viral Season

**DOI:** 10.1001/jamanetworkopen.2025.33943

**Published:** 2025-09-26

**Authors:** Nadir Ijaz, Constantin Radu, Craig Rothenberg, Alexander T. Janke, Arjun K. Venkatesh

**Affiliations:** 1 National Clinician Scholars Program, Yale University School of Medicine, New Haven, Connecticut; 2 Section of Critical Care, Department of Pediatrics, Yale University School of Medicine, New Haven, Connecticut; 3 Yale University School of Public Health, New Haven, Connecticut; 4 Department of Emergency Medicine, Yale University School of Medicine, New Haven, Connecticut; 5 Ann Arbor Veterans Affairs Healthcare System, Ann Arbor, Michigan; 6 Department of Emergency Medicine, University of Michigan, Ann Arbor; 7 Institute for Healthcare Policy and Innovation, University of Michigan, Ann Arbor; 8 Center for Outcomes Research and Evaluation, Yale University, New Haven, Connecticut

## Abstract

**Importance:**

During the 2022 respiratory viral surge, anecdotal evidence suggested that pediatric bed strain and load imbalance (hospitals in the same region experiencing disparate occupancy rates) may be linked to pediatric bed capacity changes during the preceding decade.

**Objectives:**

To determine the prevalence of pediatric bed strain and load imbalance by hospital referral region (HRR) and to evaluate the association between 2010-2021 pediatric bed capacity changes and bed strain and load imbalance during the 2022 respiratory viral season.

**Design, Setting, and Participants:**

This cross-sectional analysis of weekly mean bed strain and load imbalance from September 30, 2022, through January 5, 2023, used US Department of Health and Human Services data; longitudinal analysis of 2010-2021 pediatric bed capacity used American Hospital Association survey data. A total of 254 HRRs throughout the US were included. For bed strain analyses, inclusion criteria consisted of having at least 1 pediatric acute care bed; for load imbalance analyses, at least 2 hospitals, each with at least 1 pediatric acute care bed. Hospitals with missing data for at least 2 consecutive weeks in the study period were excluded. Data were analyzed from February 7, 2023, to July 2, 2025.

**Main Outcomes and Measures:**

Bed strain, defined as more than 85% of pediatric acute care beds in the HRR occupied, and load imbalance, defined as at least 1 hospital with at least 85% pediatric beds occupied, at least 1 hospital with less than 85% pediatric beds occupied, and a difference of at least 20% in pediatric bed occupancy between hospitals with the highest and lowest occupancies in the HRR.

**Results:**

Of 254 HRRs included in the analysis, a mean of 112 (44.1%) experienced pediatric bed strain per week. Of 123 HRRs with at least 2 hospitals with pediatric beds, a mean of 82 (66.7%) experienced pediatric load imbalance per week. In the 254 HRRs included in the analysis of pediatric bed strain, the number of pediatric beds per 10 000 children decreased from 4.57 in 2010 to 4.02 beds (12.0% decrease) in 2021. There was no association between 2010-2021 HRR-level pediatric bed capacity changes and 2022 pediatric bed strain or load imbalance.

**Conclusions and Relevance:**

In this cross-sectional analysis of the 2022 respiratory viral season, bed strain and load imbalance were common but not associated with declines in pediatric bed capacity since 2010. Regionalization efforts may prevent capacity overload and load imbalance during future surges.

## Key Points

**Question:**

During the 2022 respiratory viral season, did hospital referral regions experience pediatric bed strain and load imbalance?

**Findings:**

In this cross-sectional study of 254 hospital referral regions, a mean of 112 (44%) experienced bed strain (>85% bed occupancy) per week. In 123 regions with 2 or more hospitals with pediatric beds, a mean of 82 (67%) per week experienced load imbalance (≥1 hospital with ≥85% beds occupied, ≥1 hospital with <85% beds occupied, and ≥20% difference in bed occupancy between hospitals).

**Meaning:**

These findings suggest that load imbalance was common during the 2022 respiratory viral season; load-balancing strategies may prevent bed capacity overload in future pediatric surges.

## Teaser

This cross-sectional study describes the prevalence of pediatric bed strain and load imbalance during the 2022 respiratory viral season and evaluates the association between pediatric bed capacity changes from 2010 to 2021 and findings during the 2022 respiratory viral season by hospital referral region.

## Introduction

The 2022 respiratory viral season brought a surge in children hospitalized with respiratory syncytial virus, influenza, and SARS-CoV-2 and pediatric bed strain—reported pediatric inpatient bed shortages—throughout the US.^1^ Pediatric bed strain has been associated with increased inpatient lengths of stay,^2,3^ adverse events,^4^ and mortality,^5,6^ making it important to understand the underlying causes. Anecdotal reports linked 2022 pediatric bed strain to reductions in pediatric inpatient beds during the preceding decade.^7^ Indeed, the number of pediatric inpatient beds in the US decreased by 19.5% from 2008 to 2022, disproportionately impacting some geographic regions more than others.^8,9^ However, the association between these reductions in pediatric bed capacity and 2022 pediatric bed strain have not been explored beyond anecdotes and media statements.^7^

It is equally important to evaluate potential strategies to reduce pediatric bed strain and mitigate the downstream effects of hospital capacity overload on patient experience and outcomes. During the 2022 surge, some states used centralized coordination to balance pediatric load among hospitals within the same geographic region (a load-balancing strategy) to reduce hospital-level bed strain and mitigate these potential downstream effects.^10^ While this is a potentially impactful strategy, it would work best in situations of load imbalance, in which some hospitals experience bed strain while others do not in the same geographic area. However, no studies, to our knowledge, have measured pediatric load imbalance during the 2022 surge.

In this study, we aimed to fill these knowledge gaps with the following specific objectives: (1) to describe the prevalence of pediatric bed strain and pediatric load imbalance during the 2022 respiratory viral season in different US geographic regions by bed-to-child ratio quartile, (2) to describe the prevalence of pediatric intensive care unit (PICU) bed strain and PICU load imbalance during the 2022 respiratory viral season, and (3) to evaluate the association between pediatric bed capacity changes from 2010 to 2021 and bed strain and load imbalance during the 2022 respiratory viral season by region. We hypothesized that regions with greater reductions in pediatric bed capacity from 2010 to 2021 would have greater bed strain and load imbalance during the 2022 respiratory viral season.

## Methods

### Study Design

We conducted a cross-sectional analysis of bed strain and load imbalance during the 2022 respiratory viral season (identified as September 30, 2022, through January 5, 2023) for pediatric and PICU beds in the US. We separately conducted a longitudinal analysis of 2010-2021 pediatric bed capacity. Analyses were conducted at the level of hospital referral regions (HRRs).^11^ This study did not require institutional review board approval because it did not meet criteria for human subjects research as defined by Department of Health and Human Services regulations because it involved only publicly available data with no identifiers. This study was reported according to the Strengthening the Reporting of Observational Studies in Epidemiology (STROBE) guidelines for cross-sectional studies.

### Data Sources

We obtained bed counts and hospital location for all short-term acute care hospitals with bed counts available for any year before 2015 and any year after 2016 from the 2010-2021 American Hospital Association (AHA) Annual Survey. We calculated bed-to-child ratios for each HRR using the zip code–level population of children aged 0 to 17 years from the US Census Bureau 5-Year American Community Survey.^12^ For each HRR, we calculated the change in population-adjusted pediatric bed capacity from 2010 to 2021 as the difference between the earliest and the latest year with available data. We divided HRRs into 4 quartiles based on their bed-to-child ratio in the most recently available year. We did not impute missing bed count data.

We obtained hospital-level pediatric bed occupancy data for the 2022 respiratory viral season from the US Department of Health and Human Services (DHHS) Protect Public Data Hub.^13^ This dataset contains hospital-reported, 7-day means for pediatric and PICU bed occupancy from April 28, 2022, through February 2, 2023. Where necessary, we imputed a maximum of 1 week of missing data for the 14-week study period using the mean of the values before and after the missing value.

We converted hospital- and zip code–level data to HRR-level data using the Geocorr 2022 Geographic Correspondence Engine.^14^ The *Dartmouth Atlas of Health Care in the United States* used Medicare data to define 306 HRRs in the US, each denoting a health care market for tertiary medical care for adults requiring major cardiovascular or neurosurgical procedures.^11^ While HRRs are not specific for pediatric care, in the absence of a similar national atlas defining pediatric health care markets, we chose to conduct our analysis at the HRR level, similar to another study analyzing pediatric care patterns by geography.^15^

### Inclusion and Exclusion Criteria

We included all HRRs with at least 1 short-term acute care hospital with at least 1 pediatric bed (for pediatric strain analyses) or at least 1 PICU bed (for PICU strain analyses) reported in the most recently available AHA Annual Survey. Because our definition for load imbalance required at least 2 hospitals per HRR, for our analyses of load imbalance we included only HRRs that had at least 2 hospitals with at least 1 pediatric or PICU bed (for pediatric and PICU analyses, respectively). For all analyses, we excluded acute long-term care hospitals and hospitals limited to caring for individuals with psychiatric conditions, intellectual disabilities, rehabilitation needs, chronic diseases, and substance use disorders. We excluded hospitals with missing data for 2 or more weeks during our 14-week study period.

### Outcome Measures

Using DHHS data, we defined HRR-level pediatric or PICU bed strain as the condition in which the proportion of pediatric or PICU beds that are occupied in an HRR (as a fraction of all pediatric or PICU beds within an HRR) is greater than 85%. We chose 85% occupancy as the cutoff as higher occupancy has been associated with increased emergency department boarding,^16^ which may be an upstream driver of the negative outcomes associated with bed strain.

Similar to a prior study that examined adult critical care load imbalance during the COVID-19 surge,^17^ we defined HRR-level pediatric and PICU load imbalance as the condition under which all of the following criteria were met: (1) at least 1 hospital in the HRR had at least 85% of available pediatric or PICU beds occupied, (2) at least 1 hospital in the HRR had less than 85% of pediatric or PICU beds occupied, and (3) there was a difference of at least 20% in the occupancy of pediatric or PICU beds between the hospitals with the highest and lowest occupancies within the same HRR.

### Statistical Analysis

Data were analyzed from February 7, 2023, to July 2, 2025. We calculated the mean percentage of HRRs experiencing bed strain and load imbalance per week during the study period. We estimated the prevalence of load imbalance by geographic region and identified the HRRs experiencing bed strain and load imbalance for the greatest number of weeks during the study period. We divided HRRs into quartiles based on the proportion of weeks with (1) pediatric bed strain and (2) load imbalance to describe the geographic distributions of these 2 outcomes. To assess whether current geographic differences in bed capacity may influence our outcome variables, we also divided HRRs into separate quartiles by bed-to-child ratio and described the mean proportion of weeks with bed strain and load imbalance for each bed-to-child ratio quartile.

We then described absolute changes in pediatric and PICU bed capacity per 10 000 children aged 0 to 17 years by HRR from 2010 to 2021 using descriptive statistics. We used the Spearman correlation coefficient to evaluate the association between 2010-2021 HRR-level changes in pediatric bed capacity and the proportion of weeks HRRs experienced each of our 4 outcome measures (pediatric bed strain, PICU bed strain, pediatric load imbalance, and PICU load imbalance). We used bootstrapping to calculate 95% CIs for the Spearman correlation coefficients. We also conducted a post hoc analysis to determine the power of our sample to detect an association between 2010-2021 pediatric bed capacity changes and each of our 4 outcome measures.

We performed sensitivity analyses to determine whether altering our definition of load imbalance or excluding hospitals with low pediatric bed counts would affect our results. We did this by: (1) using a difference in percentage occupancy of at least 30% (instead of 20%) between hospitals with highest and lowest occupancy in an HRR as a cutoff for load imbalance and (2) excluding all hospitals with less than 5 pediatric beds. We performed a third sensitivity analysis to confirm that our imputation methods did not change our results by excluding all hospitals with any missing data during any week.

We used a 2-sided *P* < .05 to determine statistical significance. All analyses were performed using R Statistical Software, version 4.2.2 (R Core Team).

## Results

### Pediatric Bed Strain and Load Imbalance

Of 745 hospitals with both AHA bed count data for our years of interest and DHHS pediatric bed occupancy data for our study period, we excluded 191 hospitals due to at least 2 weeks of missing bed occupancy data and 7 for missing data in the first or last week of the study period. We therefore included 254 HRRs with 547 hospitals in our pediatric bed strain analysis (eFigure and eTable 1 in Supplement 1).

**Figure 1.**
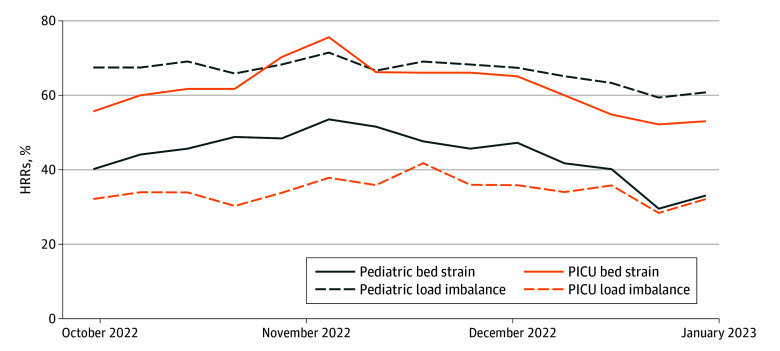
Proportion of Hospital Referral Regions (HRRs) Experiencing Pediatric and Pediatric Intensive Care Unit (PICU) Bed Strain and Load Imbalance Data include the 14-week respiratory viral surge. Analysis includes 254 HRRs for pediatric bed strain, 123 for pediatric load imbalance, 115 for PICU bed strain, and 53 for PICU load imbalance.

The median number of hospitals with at least 1 pediatric bed per HRR was 1.0 (IQR, 1.0-2.8), and the median number of pediatric beds per HRR was 53.0 (IQR, 18.0-126.8). Of the 254 HRRs included in our analysis, 159 (62.6%) experienced at least 1 week of pediatric bed strain. A mean of 112 HRRs (44.1%) experienced pediatric bed strain in any given week during the study period; 35 (13.8%) experienced pediatric bed strain in all 14 weeks. The week of November 4 to 10, 2022, had the highest number of HRRs experiencing pediatric bed strain (136 of 254 [53.5%]); the week of December 23 to 29, 2022, had the lowest number (75 of 254 [29.5%]) (Figure 1).

Of 123 HRRs with at least 2 hospitals with pediatric beds, 108 (87.8%) experienced at least 1 week of pediatric load imbalance during the study period. A mean of 82 HRRs (66.7%) experienced pediatric load imbalance in any given week during the study period; 49 (39.8%) experienced pediatric load imbalance in all 14 weeks. The proportion of HRRs experiencing pediatric load imbalance ranged from 73 of 123 (59.3%) to 88 of 123 (71.5%), peaking in the week of November 4 to 10, 2022 (Figure 1).

### PICU Strain and Load Imbalance

There were 115 HRRs that met our inclusion criteria for the PICU bed strain analysis. The median number of hospitals with at least 1 PICU bed per HRR was 1.0 (IQR. 1.0-2.0) and the median number of PICU beds per HRR was 11.0 (IQR, 8.0-18.5). Of 115 HRRs with at least 1 hospital with at least 1 PICU bed, 96 (83.5%) experienced at least 1 week of PICU bed strain. A mean of 71 of 115 HRRs (61.7%) experienced PICU bed strain in any given week during the study period; 32 (27.8%) experienced PICU bed strain in all 14 weeks. The week of November 4 to 10, 2022, had the highest number of HRRs experiencing PICU bed strain (87 of 115 [75.7%); the week of December 23 to 29, 2022, had the lowest number (60 of 115 [52.2%]) (Figure 1).

Of 53 HRRs with at least 2 hospitals with PICU beds, 49 (92.5%) experienced at least 1 week of PICU load imbalance during the study period. A mean of 18 of 53 HRRs (34.0%) experienced PICU load imbalance in any given week during the study period; 9 (17.0%) experienced load imbalance in all 14 weeks. The proportion of HRRs experiencing PICU load imbalance ranged from 15 (28.3%) to 22 (41.5%), peaking in the week of November 18 to 24, 2022 (Figure 1).

### Proportion of Weeks With Study Outcomes

**Figure 2.**
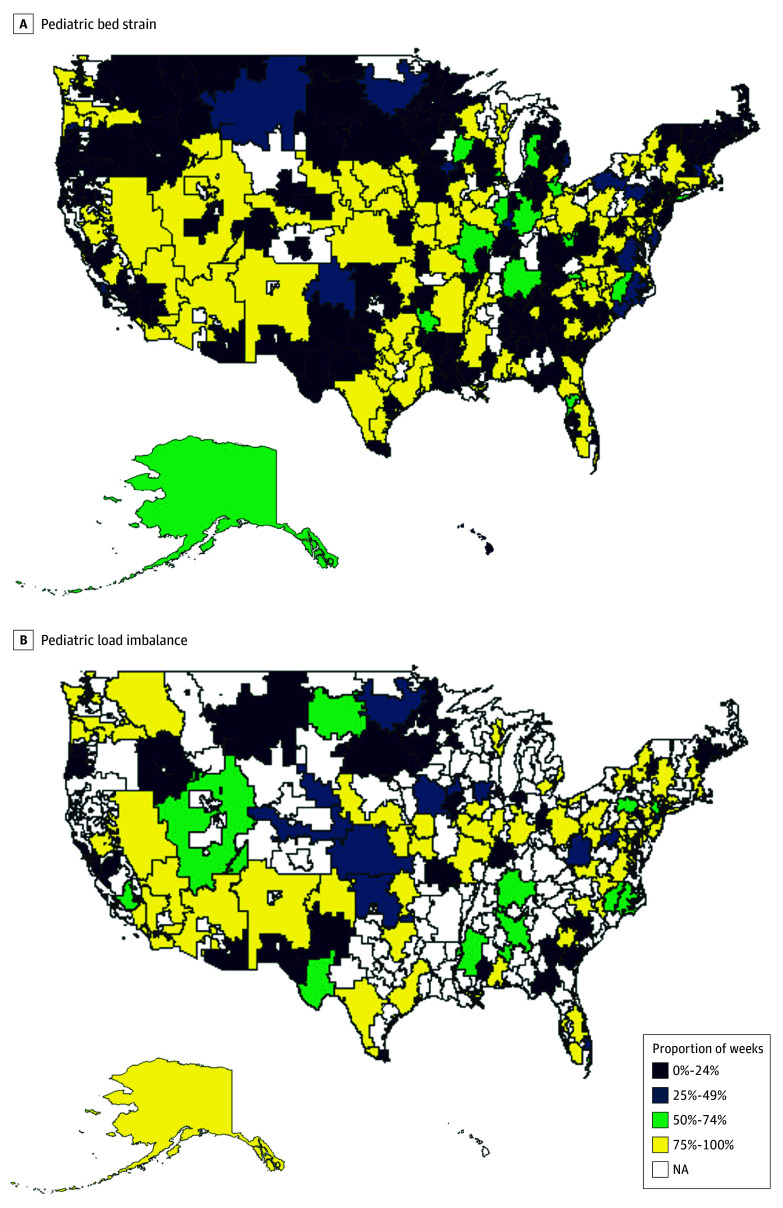
Hospital Referral Regions Depicted in Quartiles Based on Proportion of Weeks Experiencing Pediatric Bed Strain and Pediatric Load Imbalance NA indicates not included in analysis.

Table 1 shows the mean proportions of weeks with pediatric bed strain, pediatric load imbalance, PICU bed strain, and PICU load imbalance by HRR bed-to-child ratio quartile. Figure 2 shows HRRs in quartiles of proportions of weeks with pediatric bed strain and pediatric load imbalance.

**Table 1.** Mean Proportion of Weeks With Study Outcomes

Bed-to-child ratio quartile	Mean proportion of weeks, %			
	Pediatric beds		PICU beds	
	Bed strain	Load imbalance	Bed strain	Load imbalance
First (lowest)	34.5	50.0	68.3	17.0
Second	45.0	71.4	63.0	27.8
Third	40.2	61.2	46.6	30.2
Fourth (highest)	56.8	72.5	82.0	38.8

Abbreviation: PICU, pediatric intensive care unit.

### Sensitivity Analyses

Sensitivity analyses for pediatric load imbalance are presented in Table 2. We report the frequencies of each criterion in our definition of pediatric load imbalance occurring alone or in pairs, without meeting the full definition, in eTable 2 in Supplement 1.

**Table 2.** Sensitivity Analyses for Pediatric Load Imbalance

Included HRRs	Main analysis, No. (%) (n = 123)	Sensitivity analysis, No. (%) of HRRs		
		30% Difference in occupancy between maximum and minimum occupancy hospital (n = 123)	Excluding hospitals with <5 pediatric beds (n = 116)	Excluding hospitals with any missing data in any week (no imputation) (n = 44)
≥1 wk of Pediatric load imbalance	108 (87.8)	106 (86.2)	99 (85.3)	35 (79.5)
Peak with load imbalance	88 (71.5)	84 (68.3)	81 (69.8)	28 (63.6)

Abbreviation: HRR, hospital referral region.

### Association With 2010-2021 Bed Capacity Changes

In the 254 HRRs included in our analysis of pediatric strain and load imbalance, the number of pediatric beds per 10 000 children decreased from 4.57 in 2010 to 4.02 beds (a 12.0% decrease) in 2021. In the 115 HRRs included in our analysis of PICU strain and load imbalance, the number of PICU beds per 10 000 children increased from 0.69 in 2010 to 0.80 in 2021 (a 15.9% increase).

Spearman correlation coefficients between 2010-2021 pediatric and PICU bed changes and the proportion of weeks HRRs experienced each of our 4 outcomes (pediatric strain, pediatric load imbalance, PICU strain, and PICU load imbalance) are shown in Table 3. None of the tested correlations were statistically significant.

**Table 3.** Spearman Correlations for 2010-2021 Pediatric and PICU Bed Changes and Main Outcomes

Outcome	Spearman correlation coefficient (95% CI)	*P* value^a^
2010-2021 Pediatric bed changes		
Bed strain	−0.041 (−0.158 to 0.083)	.52
Load imbalance	−0.105 (−0.275 to 0.075)	.27
2010-2021 PICU bed changes		
Bed strain	−0.016 (−0.203 to 0.178)	.86
Load imbalance	−0.089 (−0.282 to 0.109)	.37

Abbreviation: PICU, pediatric intensive care unit.

^a^ Calculated using algorithm AS 89 for n < 1290; otherwise, the asymptotic t approximation was used.^18^ *P* < .05 was considered significant.

## Discussion

In this cross-sectional analysis of a pediatric bed occupancy dataset during the 2022 respiratory viral season, pediatric bed strain, pediatric load imbalance, PICU bed strain, and PICU load imbalance were common across US HRRs. Linking these data to a longitudinal analysis of a 2010-2021 pediatric bed capacity dataset, we were unable to reject the null hypothesis and found that bed capacity declines between 2010 and 2021 did not explain geographic variation in the prevalence of pediatric bed strain, pediatric load imbalance, PICU bed strain, or PICU load imbalance during the 2022 respiratory viral season.

In our study, two-thirds of HRRs experienced pediatric load imbalance in any given week during the 2022 respiratory viral surge. This finding suggests that surge strategies to balance load across hospitals equipped to provide pediatric services may be especially effective. Indeed, Washington and Oregon successfully used a load-balancing strategy and were able to change the disposition of 38% of patients for whom a pediatric intensivist subject matter expert was integrated into the referral process.^10^

Approximately one-third of HRRs experienced PICU load imbalance, which is a substantially lower proportion than the two-thirds that experienced pediatric load imbalance. This may be at least partially accounted for by a higher PICU bed strain, with almost 61.7% of HRRs experiencing PICU bed strain per week, compared to the 44.1% per week experiencing pediatric bed strain. This finding suggests that more PICUs within the same geographic region were operating close to their maximum bed capacity during the 2022 respiratory viral surge. This implies that load-balancing strategies for children requiring critical care services during surges may be less effective than for children requiring non-PICU care.

Interestingly, although only 34.0% of HRRs experienced PICU load imbalance in any given week, almost all (92.5%) experienced load imbalance during at least 1 week during the study period. While less pronounced, this difference also exists for pediatric load imbalance (66.7% of HRRs experienced pediatric load imbalance per week, while 87.8% experienced ≥1 week of pediatric load imbalance during the study period). These findings suggest that the geographic distribution of HRRs experiencing PICU and, to a lesser extent, pediatric load imbalance, changed substantially week to week during the surge. This week-to-week variability may be due to small numbers of PICU beds per HRR or may indicate that fluctuating interventions to address capacity constraints are effective. Future studies should ascertain predictors of pediatric and PICU load imbalance to identify geographic regions that would benefit most from load-balancing strategies at different times during a surge to guide future policy decisions.

Notably, the prevalence of both pediatric and PICU bed strain and load imbalance in our study is much higher than the 13.3% and 5.6% of HRRs that experienced adult ICU bed strain and load imbalance per week during the SARS-CoV-2 pandemic (July 2020 to February 2022).^17^ It is possible that this difference in the experiences of pediatric and adult ICUs during surges is due to the closure of 29.9% of pediatric inpatient units and only 4.4% of adult units from 2008 through 2022.^8^ While we did not detect an association between 2010-2021 PICU bed capacity changes and 2022 PICU bed strain or load imbalance, this may be because almost all states experienced pediatric bed closures from 2008 to 2018, with insufficient variation in bed changes across HRRs to detect associations.^9^ HRRs likely also have pediatric units and PICUs that provide different levels of pediatric general medical-surgical and critical care services, resulting in sicker patients being preferentially admitted or transferred to higher levels of care. While researchers and professional organizations such as the American Academy of Pediatrics have attempted to categorize pediatric hospital- and PICU-based services by level,^19,20^ evidence and guidance regarding which children are best treated at which level of care are lacking. Future studies should evaluate how hospital and prehospital teams make decisions regarding patient transfers in regions with multiple hospitals that provide pediatric care services to better understand whether these dynamics contribute to the higher overall bed strain and load imbalance in our study compared with the adult literature.

### Limitations

It is important to interpret our findings in the context of the following limitations. First, we chose the HRR as our geographic unit of analysis, although HRRs are based on referral patterns using Medicare data for adult patients. While others have developed pediatric acute care HRRs for a few states,^21^ including regions in all states was an important component of our analysis. Second, due to our definition of load imbalance, we excluded HRRs with only 1 hospital with pediatric beds for our analyses of load imbalance. Our analysis may therefore be less applicable to load balancing across larger geographic regions. Third, we know some regions used load-balancing strategies for pediatric patients during the study period,^10^ which may have influenced their prevalence of load imbalance. However, to our knowledge, there is no reliable resource for identifying which regions used such strategies. We were unable to control for this. Fourth, DHHS data reports weekly means, so we were unable to detect the occurrence of bed strain or load imbalance within periods shorter than a week. Fifth, our study may be underpowered to detect an association between bed strain or load imbalance and 2010-2021 bed capacity changes; a post hoc power analysis demonstrated a power of less than 0.3 to detect an association with each of our 4 outcomes. Last, we used AHA data to establish which hospitals had pediatric beds before 2022. However, there were hospitals reporting no pediatric beds in the AHA data that reported occupied pediatric beds in the DHHS data. These hospitals were excluded from our analysis, which may have resulted in underestimation of bed strain and load imbalance. Additionally, AHA data do not identify hospital closures, which may have resulted in underestimating bed capacity changes.

## Conclusions

In this cross-sectional study, we found a high overall prevalence of both pediatric and PICU bed strain and load imbalance during the 2022 respiratory viral season that was not associated with 2010-2021 pediatric bed capacity changes. Our results suggest substantial opportunity for targeted load-balancing strategies during future pediatric hospitalization surges to be effective at reducing pediatric bed strain and harm.

## Notes

Supplement 1. **eFigure.** Reasons for Hospital Exclusion From Pediatric Bed Strain Analysis **eTable 1.** Hospital Referral Region (HRR) Characteristics (n = 254) **eTable 2.** Frequencies of Pediatric Load Imbalance Criteria, Calculated Weekly at the Level of Hospital Referral Region (HRR) During the 14-Week Study Period

Supplement 2.
Data Sharing Statement
