# Supplementary material for: Pediatric Bed Capacity, Bed Strain, and Load Imbalance During the 2022 Respiratory Viral Season
Source: JAMA Netw Open. 2025 Sep 26;8(9):e2533943. doi: 10.1001/jamanetworkopen.2025.33943 (PMC12475943; doi:10.1001/jamanetworkopen.2025.33943)
Supplement: Supplement 1. — eFigure. Reasons for Hospital Exclusion From Pediatric Bed Strain Analysis eTable 1. Hospital Referral Region (HRR) Characteristics (n = 254) eTable 2. Frequencies of Pediatric Load Imbalance Criteria, Calculated Weekly at the Level of Hospital Referral Region (HRR) During the 14-Week Study Period [file jamanetwopen-e2533943-s001.pdf]

Supplementary Online Content

Ijaz N, Radu C, Rothenberg C, Janke AT, Venkatesh AK. Pediatric bed capacity, bed strain, and load imbalance during the 2022 respiratory viral season. *JAMA Netw Open.* 2025;8(9):e2533943. doi:10.1001/jamanetworkopen.2025.33943

eFigure. Reasons for Hospital Exclusion From Pediatric Bed Strain Analysis
eTable 1. Hospital Referral Region (HRR) Characteristics (n = 254)
eTable 2. Frequencies of Pediatric Load Imbalance Criteria, Calculated Weekly at the Level of Hospital Referral Region (HRR) During the 14-Week Study Period

This supplementary material has been provided by the authors to give readers additional information about their work.

© 2025 Ijaz N et al. *JAMA Network Open*.

eFigure. Reasons for Hospital Exclusion From Pediatric Bed Strain Analysis

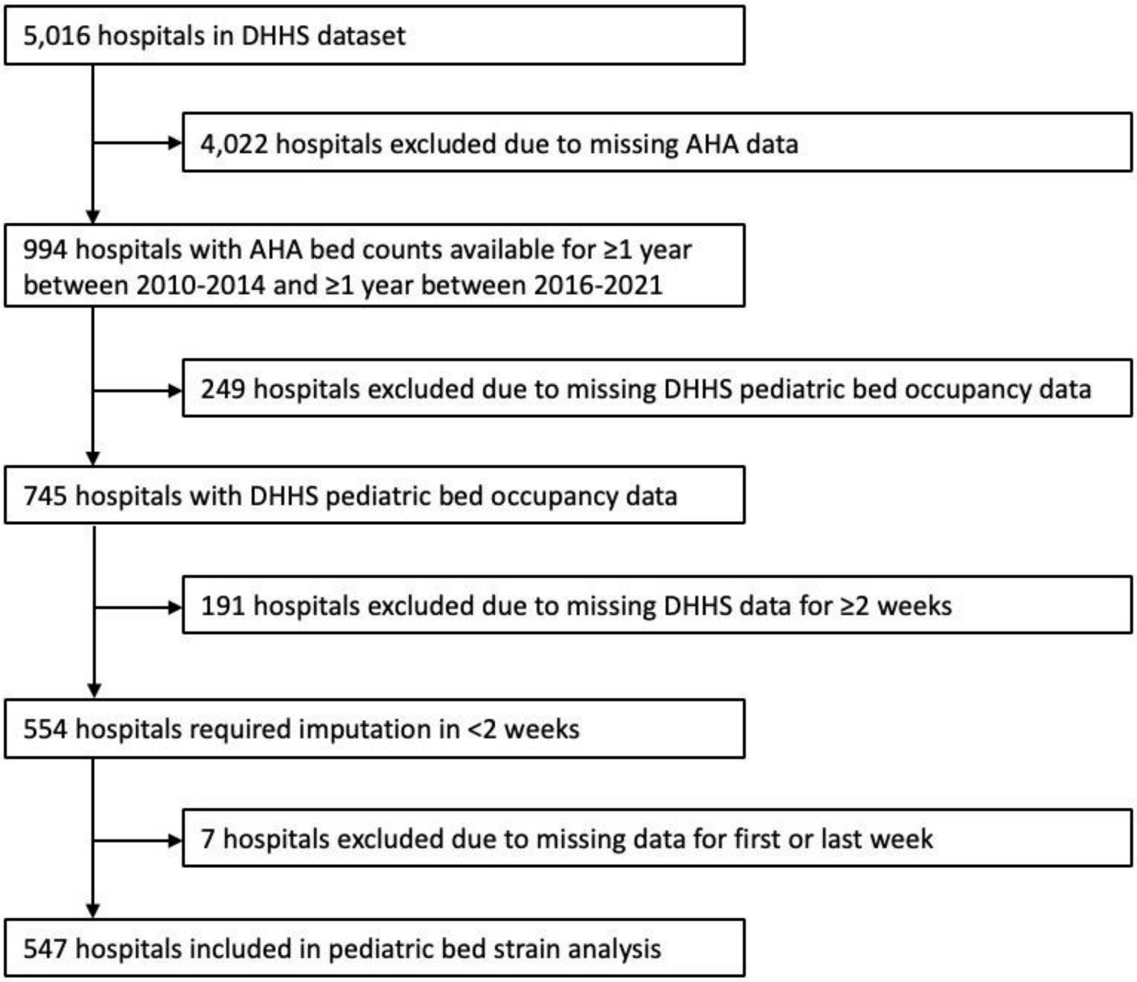

© 2025 Ijaz N et al. *JAMA Network Open*.

eTable 1. Hospital Referral Region (HRR) Characteristics (n = 254)

**Hospitals with ≥1 pediatric bed, n (%)**	
1	131 (51.6)
2	59 (23.2)
3	27 (10.6)
4	20 (7.9)
5 or more	17 (6.7)
**Geographical region, n (%)**	
Midwest	66 (26.0)
Northeast	38 (15.0)
South	101 (39.8)
West	49 (19.3)

© 2025 Ijaz N et al. *JAMA Network Open*.

eTable 2. Frequencies of Pediatric Load Imbalance Criteria, Calculated Weekly at the Level of Hospital Referral Region (HRR) During the 14-Week Study Period

**Load imbalance criteria met**	n
1. Highest occupancy hospital ≥85% only	22
2. Lowest occupancy hospital <85% only	152
3. Difference >20% only	0
1 and 2	14
1 and 3	81
2 and 3	309
1, 2, and 3 (meets load imbalance definition)	1144

© 2025 Ijaz N et al. *JAMA Network Open*.
