# Supplementary material for: Pediatric Bed Capacity, Bed Strain, and Load Imbalance During the 2022 Respiratory Viral Season
Source: JAMA Netw Open. 2025 Sep 26;8(9):e2533943. doi: 10.1001/jamanetworkopen.2025.33943 (PMC12475943; doi:10.1001/jamanetworkopen.2025.33943)
Supplement: Supplement 2. — Data Sharing Statement [file jamanetwopen-e2533943-s002.pdf]

Data Sharing Statement

Ijaz. Pediatric Bed Capacity, Bed Strain, and Load Imbalance During the 2022 Respiratory Viral Season. *JAMA Netw Open*. Published September 26, 2025.
doi:10.1001/jamanetworkopen.2025.33943

Data
Data available: No

Additional Information
Explanation for why data not available: The datasets used in our study are available via the 2010-2021 American Hospital Association (AHA) Annual Survey, the U.S. Census Bureau American Community Survey, and the U.S. Department of Health & Human Services (DHHS) Protect Public Data Hub.
